# Data-Driven Policy: An Analysis of Graduate Job Waiting Time and Its Implications for Higher Education Services

**DOI:** 10.12688/f1000research.185630.1

**Published:** 2026-07-11

**Authors:** Hermawan, Benny Hutahayan, Abd Qadir Muslim, Desmita Ayu Rachmawati

**Affiliations:** 1 Faculty of Administrative Sciences, Universitas Brawijaya, Malang, East Java, 65145, Indonesia

**Keywords:** Classification, Education Service Management, Job Waiting Time, Tracer Study

## Abstract

**Background:**

Tracer study data are more valuable for institutional evaluation than for individual-level forecasting of graduate job waiting time. This study analyzes tracer study records from the Faculty of Administrative Sciences, Universitas Brawijaya, for the 2021–2023 graduating cohorts (N = 1280).

**Methods:**

Six classification models were compared to classify graduates according to whether they secured employment within six months or experienced longer waiting times, while education-related determinants of employment transition were also examined.

**Results:**

Logistic Regression showed the highest sensitivity to delayed employment, whereas Random Forest provided relatively more balanced classification performance. The absence of internship experience emerged as the strongest barrier to faster employment, followed by poor course quality and the absence of research project participation. Limited field-specific expertise and information technology capability were also associated with delayed employment, whereas stronger English proficiency and self-development were associated with faster employment transition.

**Conclusion:**

These findings imply that universities should strengthen structured work-integrated learning, competence-oriented teaching, research engagement, digital capability, and employability development. The study contributes by repositioning tracer study data as evidence for institutional diagnosis and higher education service improvement while highlighting its complementary role alongside other approaches for individual-level prediction.

## 1. Introduction

Higher education plays a central role in producing high-quality, innovative, and productive human resources that contribute to individual advancement, social development, and economic growth ( McMahon, 2009). In the context of globalization and rapid technological change, universities are increasingly expected to prepare graduates who are adaptive, innovative, and responsive to complex labor market demands ( Carnevale & Hatak, 2020; Tight, 2021; Wulandari et al., 2023). This role is closely linked to the broader function of human resource development in strengthening productivity, competitiveness, and social welfare through education that shapes character, personality, and individual capability ( Siregar et al., 2024; Yorke, 2006). The core functions of higher education in research, teaching, and public service therefore remain fundamental in supporting graduate readiness and broader societal progress ( Perkins, 1967; Lipset, 1964).

The role of modern universities has become increasingly complex because higher education institutions are required not only to provide academic knowledge but also to respond to social, economic, and labor market pressures ( Kerr, 1963). Universities need to balance specialization with interdisciplinary capability, maintain teaching quality, and ensure meaningful faculty–student interaction so that graduates are not academically isolated from employment opportunities ( Cowen, 1981; Lipset, 1964). In this respect, the quality of higher education is strongly reflected in graduates’ readiness to enter the workforce and contribute to society ( Cheng et al., 2022). Internal and external quality assurance therefore becomes essential in ensuring that institutions remain responsive to technological change and stakeholder expectations ( Kayyali, 2023). As institutions implementing the *tri dharma* (Three Pillars of Higher Education, including: teaching, research, and community service), universities are also expected to sustain graduate quality, faculty and teaching quality, and the broader continuity of academic contribution ( Ubihatun et al., 2024). In the digital era, this role becomes even more strategic because higher education must equip students with analytical thinking, creativity, and technological literacy to face global challenges.

The urgency of graduate transition into employment is increasingly visible in both regional and national contexts. Based on ILOSTAT data (2023; 2024), the Asia–Pacific region continues to face major labor market challenges despite economic growth, including a substantial jobs gap and demographic pressures associated with population aging. Declining labor force participation, persistent unemployment, and limited job creation indicate that the transition from education to employment is becoming more competitive and more difficult for many young people. In Indonesia, the number of higher education graduates reached 1,535,074 in 2023, while unemployment among diploma and bachelor’s degree graduates also increased in relative terms ( BPS, 2023). Higher education is often viewed as a form of human capital investment and a channel of social mobility, yet the available evidence indicates that graduate qualifications do not always align with labor market needs ( Adriani et al., 2019; Setyanti & Finuliyah, 2022). This mismatch contributes to prolonged job waiting time and makes university graduates vulnerable to delayed labor market absorption.

The problem is closely related to mismatch between graduate competencies and industry demand. According to Kellermann and Sagmeister (2000), mismatch may take the form of underqualification, underutilization, or overqualification, each of which hinders graduates’ transition into the labor market. Such mismatch not only prolongs the time needed to obtain first employment but also intensifies the social and economic risks associated with slow labor absorption, which Becker (1993) describes as “bubbling lava in a social volcano.” Graduate job waiting time, understood as the period between graduation and the acquisition of first employment, may range from 1–3 months, 4–6 months, 7–12 months, or more than 12 months ( Damayanti, 2018). Prior evidence also shows that waiting time differs across fields of study, with graduates in science, mathematics, and computer-related fields tending to obtain employment more quickly than graduates in the humanities and education (Qiyomiddin, 2024). These patterns indicate that job waiting time is shaped not only by labor market conditions but also by the relevance of graduate competencies and educational preparation.

From the perspective of higher education, the Outcome-Based Education (OBE) approach emphasizes that universities are expected to produce graduates who are ready for work and aligned with market needs ( Spady, 1994). Graduate employability therefore becomes an important indicator of how effectively higher education responds to labor market demand. High employment absorption suggests that graduate competencies are relevant, whereas prolonged waiting time signals skill gaps and possible weaknesses in educational preparation ( Deffinika et al., 2022; Utari, 2022). Universities also occupy an important position in the political–economic system because they shape ideas, policies, and future labor supply, even though they are sometimes criticized for emphasizing adaptation over transformative change ( Abrams, 1968). In the context of public education services in Indonesia, higher education is also expected to meet broader societal needs through effective, transparent, and accountable service delivery, while continuously improving educational quality in response to stakeholder expectations and social change ( Martensen et al., 2000; Peters & Waterman, 1982; Hill, 1995; Becker, 1993).

One of the main instruments for such evaluation is the tracer study. As part of the assessment of Key Performance Indicators, tracer studies function as benchmarks for higher education institutions in monitoring whether graduates are employed, continue their studies, or pursue entrepreneurship. Within the *Merdeka Belajar* (Freedom to Learn) framework, tracer studies are increasingly important for curriculum adaptation, responsiveness to industry change, and the strengthening of graduate competitiveness in the digital. The *Kampus Merdeka* (Independent Campus) policy also places emphasis on graduate absorption within six months after graduation, making job waiting time a practically significant outcome for institutional evaluation. At Universitas Brawijaya, tracer studies have consistently been used to evaluate graduate alignment with labor market needs and to support educational strategy development. Ministry data indicate that 81.8 percent of graduates work in fields aligned with their area of study, while 74.0 percent work in positions aligned with their education level, although substantial variation remains across programs within the Faculty of Administrative Sciences.

As higher education institutions accumulate larger volumes of graduate data, the potential use of analytical approaches, including data mining, has become increasingly important ( Parasuraman et al., 1988; Aprilla Dennis, 2013). Classification methods such as Naïve Bayes and Decision Tree C4.5 have been used to process complex datasets and uncover hidden patterns ( Kaithwas & Borkar, 2019; Hariri et al., 2019; Fuad et al., 2020). However, the application of predictive approaches in tracer study research remains limited and has tended to emphasize descriptive analysis rather than a systematic examination of the factors shaping graduate job waiting time ( Fuad et al., 2020; Sutadji et al., 2021). Previous studies have also more often emphasized external labor market conditions than internal education-related factors such as learning experiences and graduate competencies. Therefore, this study addresses that gap by applying comparative classification techniques to tracer study data from the Faculty of Administrative Sciences, Universitas Brawijaya, in order to examine graduate job waiting time and identify education-related factors associated with employment transition, thereby providing a stronger evidence base for data-informed higher education services and institutional efforts to accelerate graduate absorption into the labor market.

## 2. Objectives and research questions

This study aims to examine the usefulness of tracer study data for understanding graduate job waiting time and its implications for higher education services. Specifically, the study compares the performance of six classification models—Naïve Bayes, Decision Tree C4.5, Logistic Regression, Random Forest, Support Vector Machine, and Gradient Boosted Trees—in distinguishing graduates who obtained employment within six months from those with longer waiting times. In addition, the study identifies education-related factors associated with employment transition in order to clarify which dimensions of learning experience and graduate competence are most relevant to delayed or faster labor market absorption. Accordingly, this study asks whether tracer study data can meaningfully support the classification of graduate job waiting time, which of the evaluated models is relatively more suitable for identifying delayed employment, which education-related factors are associated with faster or slower transition into employment, and what these findings imply for higher education services and graduate employability support.

## 3. Literature review

### 3.1. Higher education and job readiness

Higher education plays a strategic role in preparing students for successful transition into the labor market. Beyond its academic function, higher education contributes to the development of knowledge, character, and adaptive capacity needed for graduates to respond to changing social and economic demands. In this context, graduate job readiness is not formed solely through formal instruction, but also through the broader educational environment in which students develop academic, professional, and personal competencies.

As institutions implementing the *tri dharma*, universities are expected to produce graduates who are competent, adaptive, and capable of contributing to society. This expectation places job readiness at the center of higher education effectiveness. The quality of curriculum design, academic processes, teaching practices, and students’ learning experiences influences how far graduates are prepared to enter employment. Course quality, practical activities, research involvement, discussions, and field experience provide a foundation for both hard skills and soft skills that support labor market entry ( Warsah, 2023).

Accordingly, graduate job waiting time can be understood as one indicator of how effectively higher education supports labor market transition. A shorter waiting time suggests that graduates possess competencies and experiences that are relevant to employment demands, whereas longer waiting time may reflect weaker preparation, limited experience, or misalignment between educational processes and labor market needs. In this sense, higher education and job readiness are closely connected through the institutional capacity to create meaningful learning experiences and to prepare graduates for employment transition.

### 3.2. Outcome-based education (OBE) and graduate competence

OBE places learning outcomes at the center of curriculum design, teaching, and evaluation. Introduced as a response to labor market demands for measurable and applicable graduate competencies, OBE emphasizes that educational success is determined not merely by instructional delivery, but by the extent to which students achieve clearly defined outcomes ( Spady, 1994). In higher education, this perspective is particularly relevant because labor market transition increasingly depends on whether graduates possess competencies that are visible, relevant, and recognized by employers.

Operationally, OBE links intended learning outcomes with Program Outcomes and Course Outcomes that guide curriculum content, learning activities, and assessment. This framework ensures that higher education is oriented not only toward knowledge acquisition but also toward the development of technical competence, critical thinking, communication, teamwork, and professionalism ( Biggs & Tang, 2020). As such, OBE provides a structured way to align institutional goals, curriculum design, and student capability development with labor market expectations.

The framework proposed by McNeir (1993) further emphasizes that stakeholder input, institutional mission, program objectives, and learning outcomes must be integrated with continuous improvement. In the context of this study, such an approach is relevant because tracer study variables—such as internship experience, course quality, field-specific competence, information technology skills, soft skills, and engagement in academic activities—can be interpreted as manifestations of graduate competence shaped by outcome-oriented learning. Graduate job waiting time therefore serves as an external indicator of how far these competencies are translated into labor market recognition.

### 3.3. Employability framework

Employability is a multidimensional concept that explains graduates’ ability to obtain and retain employment through the interaction of knowledge, skills, experience, and personal attributes. In higher education, employability is not the product of a single factor, but of the combined influence of academic capital, soft skills, practical learning, and career readiness. This framework is particularly useful for explaining why graduate job waiting time varies, even among individuals with similar formal educational backgrounds.

From the perspective of Human Capital Theory, education enhances individual productivity through the accumulation of knowledge, intellectual ability, and technical skill ( Becker, 1993). In this study, field-specific competence, information technology literacy, and the quality of academic processes represent forms of academic capital that may strengthen graduate transition into employment. However, employability also depends on non-technical abilities. Communication, ethics, teamwork, adaptability, and problem solving are increasingly valued because employers assess not only academic achievement but also graduates’ interpersonal and professional capability ( Andrews & Higson, 2008; Alt & Raichel, 2022).

Practical learning further strengthens employability. The Work-Integrated Learning approach emphasizes that internships, field projects, and professional practice help students connect theory with workplace expectations and develop greater readiness for employment ( Patrick et al., 2008). This process is reinforced by Experiential Learning, which highlights the role of experience, reflection, conceptualization, and application in competence formation ( Kolb, 1984). At the same time, career readiness involves psychological and strategic preparedness for entering employment, including self-development, digital capability, and English proficiency, which are increasingly important in contemporary labor markets. Taken together, these perspectives suggest that graduate job waiting time is shaped by the interaction of academic capital, soft skills, practical experience, and career readiness.

### 3.4. Service quality in higher education

Service quality in higher education refers to the extent to which educational processes, academic support, and institutional arrangements effectively meet student and stakeholder expectations while contributing to meaningful learning outcomes. In this context, higher education is not evaluated solely by academic output, but also by how well its services support students’ competence formation and transition into employment. Service quality models such as SERVQUAL and HEdPERF highlight that educational quality is experienced through dimensions such as reliability, responsiveness, assurance, empathy, and academic quality ( Parasuraman et al., 1988).

Within higher education, these dimensions are reflected most clearly in academic processes, teaching quality, and curriculum relevance. Academic processes include faculty–student interaction, learning methods, participation in academic activities, and opportunities for collaborative and practical learning ( De Hei et al., 2015). High-quality processes create learning environments that support analytical ability, communication, teamwork, and critical thinking. Teaching quality influences not only students’ academic achievement but also their adaptability and preparedness for professional life ( Douglas, 2015). Similarly, curriculum relevance determines whether students acquire competencies that match technological change and labor market demand.

In service management terms, these educational dimensions can be understood as core institutional mechanisms through which universities shape graduate readiness. Curriculum development, academic administration, facilities, and continuous feedback processes all contribute to the effectiveness of higher education services in preparing graduates for employment transition ( Kotler & Fox, 1995; Tjiptono & Chandra, 2016; Schneider & Bowen, 2010). In this sense, graduate job waiting time is not only an employment outcome, but also an indirect indicator of how effectively higher education services support learning, competence development, and labor market preparation.

### 3.5. Tracer study and job waiting time variables

Tracer studies are one of the main instruments used by higher education institutions to evaluate graduate outcomes and the relevance of education to labor market needs. They provide information on graduates’ employment pathways, field-of-study match, further study, and other early career outcomes, thereby helping universities assess how effectively their educational processes support post-graduation transition ( Akbar & Mukhtar, 2020; Schomburg, 2010). As an evaluative tool, tracer studies connect the input, process, output, and outcome dimensions of higher education and provide a basis for curriculum improvement and institutional reflection.

In addition to documenting outcomes, tracer studies offer strategic value for reviewing study program effectiveness, strengthening university–industry alignment, and improving workforce preparation ( Yunanto et al., 2021; Nurizzati, 2019. Variables commonly found in tracer studies—such as internship experience, course quality, field-specific competence, research participation, discussions, fieldwork, and soft skills—can be interpreted as indicators of students’ learning experiences and graduate competence. As a result, tracer studies make it possible to examine how educational processes are related to labor market transition, including graduate job waiting time.

However, tracer studies are still more often used for descriptive reporting than for systematic analytical examination of education-related determinants of labor market transition. Much existing use focuses on employment status, field relevance, or aggregate graduate outcomes, while less attention is given to how tracer study variables can be used to understand differences in job waiting time. This limitation is important because graduate transition outcomes are shaped not only by external labor market conditions but also by internal educational factors such as learning experiences, teaching quality, and competence formation. Accordingly, this study uses tracer study data not only as a reporting instrument but also as an analytical basis for examining job waiting time and identifying education-related factors relevant to higher education service improvement.

## 4. Research method

This study uses a retrospective quantitative comparative design based on multi-year tracer study data (2021–2023) with a pooled cross-sectional approach. This design is selected because each respondent is measured only once in their respective graduation year, maintaining a cross-sectional structure while capturing temporal variation across multiple years. The use of multi-year data allows for a more comprehensive analysis of graduate job waiting time dynamics while preserving a non-experimental framework. To minimize potential temporal bias arising from differences in labor market conditions across years, such as the impact of the COVID-19 pandemic and the economic recovery phase, this study includes a dummy variable for “graduation year” (2021, 2022, and 2023) in the regression model. This approach enables statistical control of year effects, thereby improving the validity and generalizability of inferential results across periods.

The research was conducted at the Faculty of Administrative Sciences, Universitas Brawijaya, located at St. MT Haryono 163, Malang, East Java, Indonesia. The tracer study covered graduates from the 2021–2023 cohorts. Tracer study data collection were conducted between 15 January 2026 and 31 January 2026 following the issuance of ethical approval on 12 January 2026. Each graduate contributed a single record linked to their respective cohort year, consistent with the pooled cross-sectional design, and all data were processed in anonymized form.

Although the tracer study data show class imbalance between the “Fast” and “Not Fast” categories, this study does not apply balancing techniques such as oversampling, undersampling, SMOTE, or class weighting. This decision is made to evaluate the natural performance of each model on the actual data distribution without sample manipulation. RapidMiner applies stratified k-fold cross-validation so that each fold preserves the same class proportions as the original dataset, but it does not perform explicit class balancing. Therefore, the results reflect the algorithms’ ability to handle imbalanced data directly.

All data mining analysis processes in this study are conducted using RapidMiner Studio version 9.10, a comprehensive and integrated data science platform. The selection of RapidMiner is based on several methodological considerations: (1) its native capability to handle the six classification algorithms used in this study without requiring manual coding, (2) built-in features for fully automated k-fold cross-validation, (3) support for simultaneous computation of comprehensive evaluation metrics, including precision, recall, F1-score, ROC–AUC, and Cohen’s Kappa, (4) intuitive model visualization that is easy for education stakeholders to interpret, and (5) flexible handling of missing data and imbalanced classes through various preprocessing operators.

### 4.1. Main model parameters

To ensure traceability and reproducibility of the modeling process, all hyperparameters used in the six classification algorithms are explicitly reported. These parameters represent the actual configurations applied during the model training phase within the cross-validation process in RapidMiner Studio. By documenting all core model settings, this study enables other researchers to replicate the results consistently or to conduct model comparisons using similar datasets. Table 1 summarizes the hyperparameter settings implemented for each classifier reflecting the exact configurations used during cross-validation in RapidMiner Studio.

**Table 1.** Hyperparameter settings of classification models used in the study.

Model	Parameter	Value	Description
**Decision Tree (C4.5)**	criterion	gain_ratio	Attribute selection based on Gain Ratio
	maximal depth	10	Maximum tree depth
	apply pruning	TRUE	Enable pruning
	confidence	0.1	Confidence factor for pruning
	apply prepruning	TRUE	Enable pre-pruning
	minimal gain	0.01	Minimum gain improvement threshold
	minimal leaf size	2	Minimum samples per leaf
	minimal size for split	4	Minimum samples required for a split
	number of prepruning alternatives	3	Number of pre-pruning alternatives
**Naïve Bayes**	laplace correction	TRUE	Avoid zero probabilities
**Logistic Regression**	early stopping	TRUE	Stop training when convergence is reached
	stopping rounds	3	Number of iterations without improvement
	stopping tolerance	0.001	Convergence threshold
	standardize	TRUE	Feature standardization
	nonnegative coefficients	FALSE	Allow negative coefficients
	add intercept	TRUE	Add intercept (constant term)
	compute pvalues	TRUE	Compute statistical significance
	remove collinear columns	TRUE	Remove multicollinearity
	missing values handling	MeanImputation	Missing value handling
	max iterations	0	Unlimited iterations
**Support Vector Machine (SVM)**	kernel type	dot	Linear kernel
	kernel cache	200	Cache size
	C	0.0	Default regularization
	convergence epsilon	0.001	Convergence threshold
	max iterations	100000	Maximum number of iterations
	scale	TRUE	Standardize features
	L pos	1.0	Positive class cost
	L neg	1.0	Negative class cost
	epsilon	0.0	Epsilon parameter
	epsilon plus	0.0	
	epsilon minus	0.0	
**Gradient Boosted Trees (GBT)**	number of trees	50	Number of trees
	reproducible	TRUE	Enable result reproducibility
	maximal depth	5	Maximum depth
	min rows	10.0	Minimum samples per node
	min split improvement	1.0E5	Minimum impurity improvement threshold
	number of bins	20	Predictor discretization
	learning rate	0.01	Boosting step size
	sample rate	1.0	Sample proportion
	distribution	AUTO	Automatic loss function
	early stopping	FALSE	Not used
	max runtime seconds	0	Not limited
**Random Forest (RF)**	number of trees	100	Number of trees
	criterion	gain_ratio	Attribute selection
	maximal depth	10	Maximum depth
	apply pruning	TRUE	Pruning enabled
	apply prepruning	TRUE	Pre-pruning enabled
	random splits	FALSE	Do not use additional random splits
	guess subset ratio	TRUE	Automatic feature subset selection
	voting strategy	confidence vote	Confidence-based voting
	enable parallel execution	TRUE	Parallel execution

### 4.2. Analysis workflow diagram

The flow diagram in Figure 1 illustrates the overall analysis process conducted in this study. The stages begin with data retrieval and preprocessing, which include data cleaning, variable transformation, and attribute role assignment. After the preprocessing stage, the analysis is divided into two main branches: (1) the job waiting time prediction branch using six machine learning algorithms—Decision Tree, Naïve Bayes, Logistic Regression, Random Forest, Gradient Boosted Trees, and Support Vector Machine—and (2) the factor analysis branch that examines determinants of job waiting time using logistic regression.

**Figure 1.**
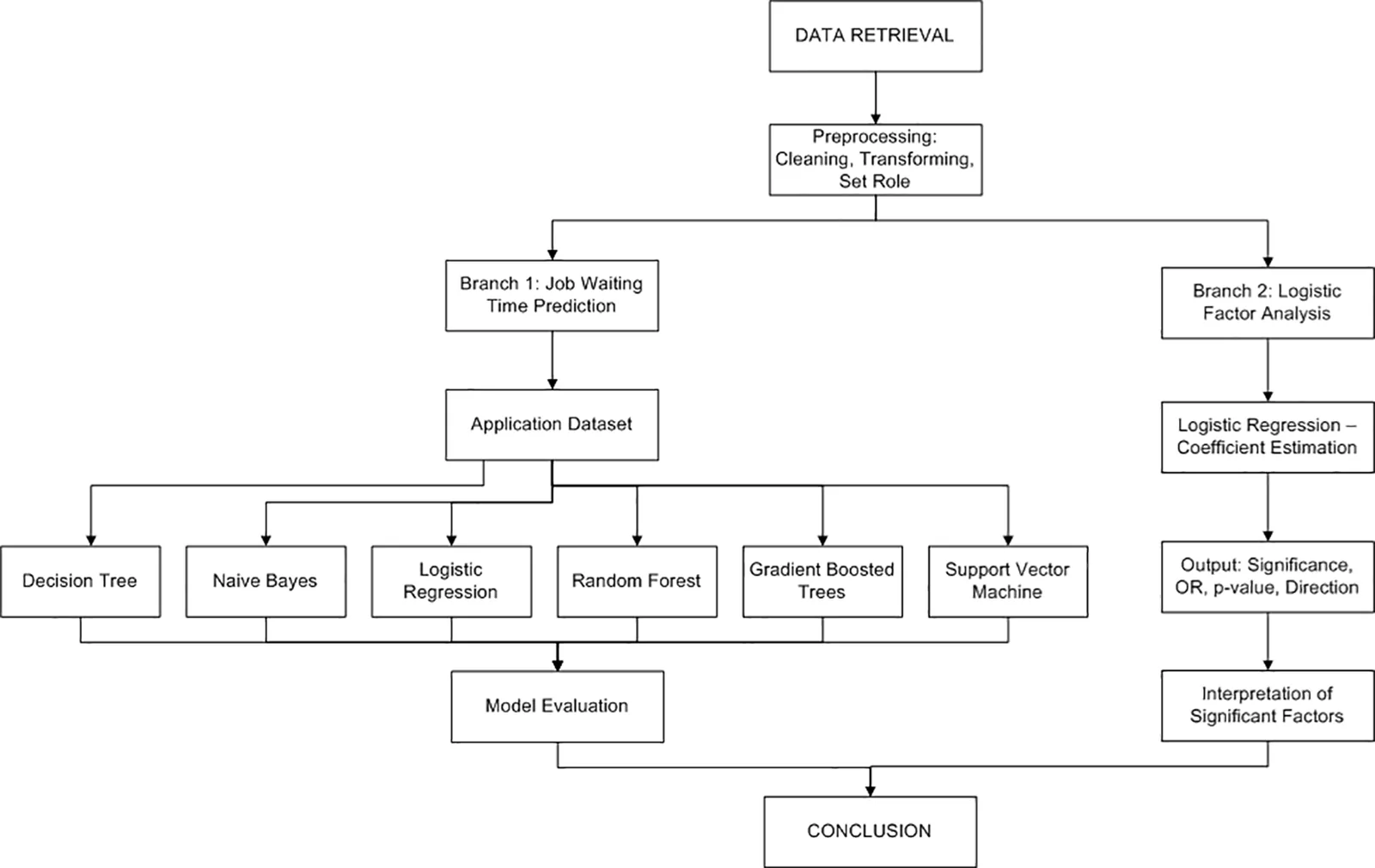
Analysis workflow for job waiting time prediction and factor identification.

### 4.3. Selection of classification methods

The selection of classification algorithms in this study is based on the characteristics of tracer study data, which mostly consist of categorical or mixed-scale variables, and on the need for interpretability to support higher education policy decisions. Naïve Bayes is selected as a baseline model because of its simplicity, robustness on medium-sized datasets, and strong performance with categorical variables. Decision Tree (C4.5) is chosen for its ability to handle both numerical and categorical variables, its tolerance for missing data, and its production of decision rules that are easy for stakeholders to interpret. To address concerns regarding result generalizability, this study includes comparative analysis using logistic regression, which is a standard inferential model in labor and employment studies, and reports comprehensive evaluation metrics, including accuracy, precision, recall, and F1-score, along with k-fold cross-validation procedures. This approach aims to combine model interpretability (Decision Tree), a simple probabilistic baseline (Naïve Bayes), and an inferential model (logistic regression), ensuring that findings do not rely on a single analytical method.

### 4.4 Research variables

The variables used in this study consist of a response variable (class) and predictor variables (attributes). The response variable is job waiting time, which is dichotomized into two categories—Fast (WT ≤ 6 months) and Not Fast (WT > 6 months)—as defined in Table 2. The predictor variables represent graduate competencies and learning-related attributes captured in the tracer study instrument. Table 3 presents the operational definitions of all predictor variables used in the modeling process.

**Table 2.** Classification of job waiting time categories.

Variable	Criteria	Category
Job Waiting Time	WT < 6 months	Fast
	WT > 6 months	Not Fast

**Table 3.** Operationalization of predictor variables.

Variable	Operational definition
Ethics	Standards of right and wrong in society that guide behavior ( Hambali et al., 2021). Ethical teachings assess good and bad actions based on utility and reason ( Wahyuningsih, 2022).
Field-Specific Expertise	Proficiency in a particular field of knowledge, including theory, concepts, and practice.
English Language Skills	Competence in reading, speaking, listening, and writing ( Karmila Sari, 2019).
Information Technology	Technology used to process, organize, and store data so that it becomes useful ( Tri Rachmadi, 2020).
Communication	The competence to understand context, message content, and nonverbal behavior ( Spitzberg & Cupach, 1984).
Teamwork	The ability to complement one another to achieve goals effectively ( Siagian, 2020; Tupti et al., 2022).
Self-Development	Continuous optimization of personal potential ( Hernowo, 2004).
Lectures (Course Instruction)	Structured methods in higher education that include academic content, thinking skills, and student attitudes ( Arikunto, 2006).
Demonstration	An effective method for teaching skills through direct observation ( Gagné, 1970).
Research Projects Participation	Active involvement in research activities, from planning to results analysis, aimed at developing students’ critical thinking and research skills.

### 4.5 Population and sample

In this study, the population consists of graduates from the Faculty of Administrative Sciences who completed their bachelor’s degree during the 2021–2023 period. The quantitative phase of this research applies a census sampling technique. In the context of data mining, census sampling is used to ensure that all relevant data that meet specific criteria are included in the analysis, with the aim of obtaining more comprehensive and representative information from the available dataset. Census sampling in this study refers to the inclusion of all data from the defined population or selected subset. The sample therefore includes all tracer study data of graduates from the Faculty of Administrative Sciences, Universitas Brawijaya, who graduated in 2021–2023, successfully completed their bachelor’s degree, and provided complete information regarding the time required to obtain their first job as recorded in the tracer study results. The distribution of the quantitative sample by study program is summarized in Table 4.

**Table 4.** Population and sample distribution by study program (2021–2023).

Year of graduation	Study program	Total records
Public Administration	2021–2023	530
Business Administration	2021–2023	750
Total		1280

Meanwhile, in the qualitative phase, the study uses purposive sampling by deliberately selecting informants based on predefined criteria derived from the quantitative results related to the tracer study. The profiles of qualitative informants and their study programs are presented in Table 5.

**Table 5.** Profile of qualitative study informants selected through purposive sampling.

Graduate cohort (Year)	Study program
Graduates of the 2021–2022 Cohort	Bachelor of Public Administration
	Bachelor of Library and Information Science
	Bachelor of Educational Administration
	Bachelor of Business Administration
	Bachelor of Tourism Administration
	Bachelor of Tax Administration

## 5. Results

### 5.1. Baseline accuracy under class imbalance

As an initial performance indicator, this section reports the classification accuracy achieved by each machine learning model under stratified k-fold cross-validation. Table 6 presents the accuracy results (mean ± standard deviation across folds, with micro-average accuracy) for the six classifiers, providing a baseline comparison of overall predictive correctness before examining more class-sensitive metrics.

**Table 6.** Classification accuracy of machine learning models.

Model	Accuracy
Naïve Bayes	84.19% +/0.91% (micro average: 84.19%)
Decision Tree	84.73% +/0.65% (micro average: 84.73%)
Logistic Regression	66.49% +/6.20% (micro average: 66.49%)
Random Forest	84.32% +/1.59% (micro average: 84.32%)
Support Vector Machine	84.73% +/0.65% (micro average: 84.73%)
Gradient Boosted Trees	72.70% +/6.77% (micro average: 72.70%)

The accuracy results show that Decision Tree and Support Vector Machine achieved the highest overall accuracy (84.73%), followed by Random Forest (84.32%) and Naïve Bayes (84.19%), while Logistic Regression and Gradient Boosted Trees produced lower accuracy scores. However, these results should be interpreted cautiously because the dataset is imbalanced, with the “Fast” employment category dominating the “Not Fast” category. Therefore, high accuracy mainly reflects the models’ tendency to classify graduates into the majority class rather than their ability to identify graduates with delayed employment. For this reason, accuracy is treated only as a preliminary indicator. The more important implication for higher education is that standard tracer study data may have limited capacity for precise individual-level prediction, but they remain useful for identifying institutional patterns related to graduate transition and employability support.

### 5.2 Comparative model evaluation under class imbalance

Given the substantial class imbalance between the “Fast” and “Not Fast” categories, accuracy alone can be misleading and may mask a model’s inability to detect the minority outcome. Therefore, this section evaluates each classifier using minority-class–focused metrics by treating “Not Fast” as the positive class. Table 7 presents the performance results for precision, recall, F1-score, Cohen’s Kappa, and ROC–AUC (reported as mean ± standard deviation across cross-validation folds where applicable), providing a more discriminative assessment of how well each model identifies graduates who take longer than six months to secure their first job.

**Table 7.** Performance evaluation of classification models on the minority class.

**Naïve Bayes**	
Precision	0.00% (positive class: Not Fast)
Recall	0.00% +/0.00% (micro average: 0.00%) (positive class: Not Fast)
F1-score	unknown (positive class: Not Fast)
kappa	0.010 +/0.013 (micro average: 0.011)
AUC	0.507 +/0.079 (micro average: 0.507) (positive class: Not Fast)
**Decision Tree**	
Precision	unknown (positive class: Not Fast)
Recall	0.00% +/0.00% (micro average: 0.00%) (positive class: Not Fast)
F1-score	unknown (positive class: Not Fast)
kappa	0.000 +/0.000 (micro average: 0.000)
AUC	0.500 +/0.000 (micro average: 0.500) (positive class: Not Fast)
**Logistic Regression**	
Precision	17.06% +/7.02% (micro average: 17.39%) (positive class: Not Fast)
Recall	31.74% +/16.06% (micro average: 31.86%) (positive class: Not Fast)
F1-score	21.72% +/9.00% (micro average: 22.50%) (positive class: Not Fast)
kappa	0.030 +/0.102 (micro average: 0.034)
AUC	0.540 +/0.107 (micro average: 0.540) (positive class: Not Fast)
**Random Forest**	
Precision	**44.17% +/30.94% (micro average: 44.00%) (positive class: Not Fast)**
Recall	9.70% +/6.54% (micro average: 9.73%) (positive class: Not Fast)
F1-score	15.94% (positive class: Not Fast)
kappa	0.107 +/0.097 (micro average: 0.110)
AUC	0.570 +/0.068 (micro average: 0.570) (positive class: Not Fast)
**Support Vector Machine**	
Precision	unknown (positive class: Not Fast)
Recall	0.00% +/0.00% (micro average: 0.00%) (positive class: Not Fast)
F1-score	unknown (positive class: Not Fast)
kappa	0.000 +/0.000 (micro average: 0.000)
AUC	0.579 +/0.104 (micro average: 0.579) (positive class: Not Fast)
**Gradient Boosted Trees**	
Precision	20.45% +/16.07% (micro average: 20.35%) (positive class: Not Fast)
Recall	18.55% (positive class: Not Fast)
F1-score	11.20% +/4.614 (micro average: 11.20%) (positive class: Not Fast)
kappa	0.025 +/0.155 (micro average: 0.023)
AUC	0.546 +/0.116 (micro average: 0.546) (positive class: Not Fast)

The minority-class evaluation shows variation in model performance in identifying graduates in the “Not Fast” category. Logistic Regression produced the highest recall, indicating better sensitivity in detecting graduates with longer job waiting time, while Random Forest recorded the highest precision and Cohen’s Kappa, indicating relatively more stable classification performance. Support Vector Machine achieved the highest AUC value, although the overall AUC range across models remained close. These results indicate that each model provides a different perspective on graduate employment transition patterns, with Logistic Regression and Random Forest offering the most useful results for further interpretation in relation to tracer study variables and higher education service evaluation.

### 5.3 ROC-based discrimination and relative model suitability

Figure 2 presents the Receiver Operating Characteristic (ROC) curves for the six classification models used to predict the “Not Fast” category as the positive class in the Job Waiting Time variable. The Area Under the Curve (AUC) value is used to measure the discriminative ability of each model in distinguishing between the “Fast” and “Not Fast” categories. A higher AUC value indicates better model performance in correctly classifying the data.

**Figure 2.**
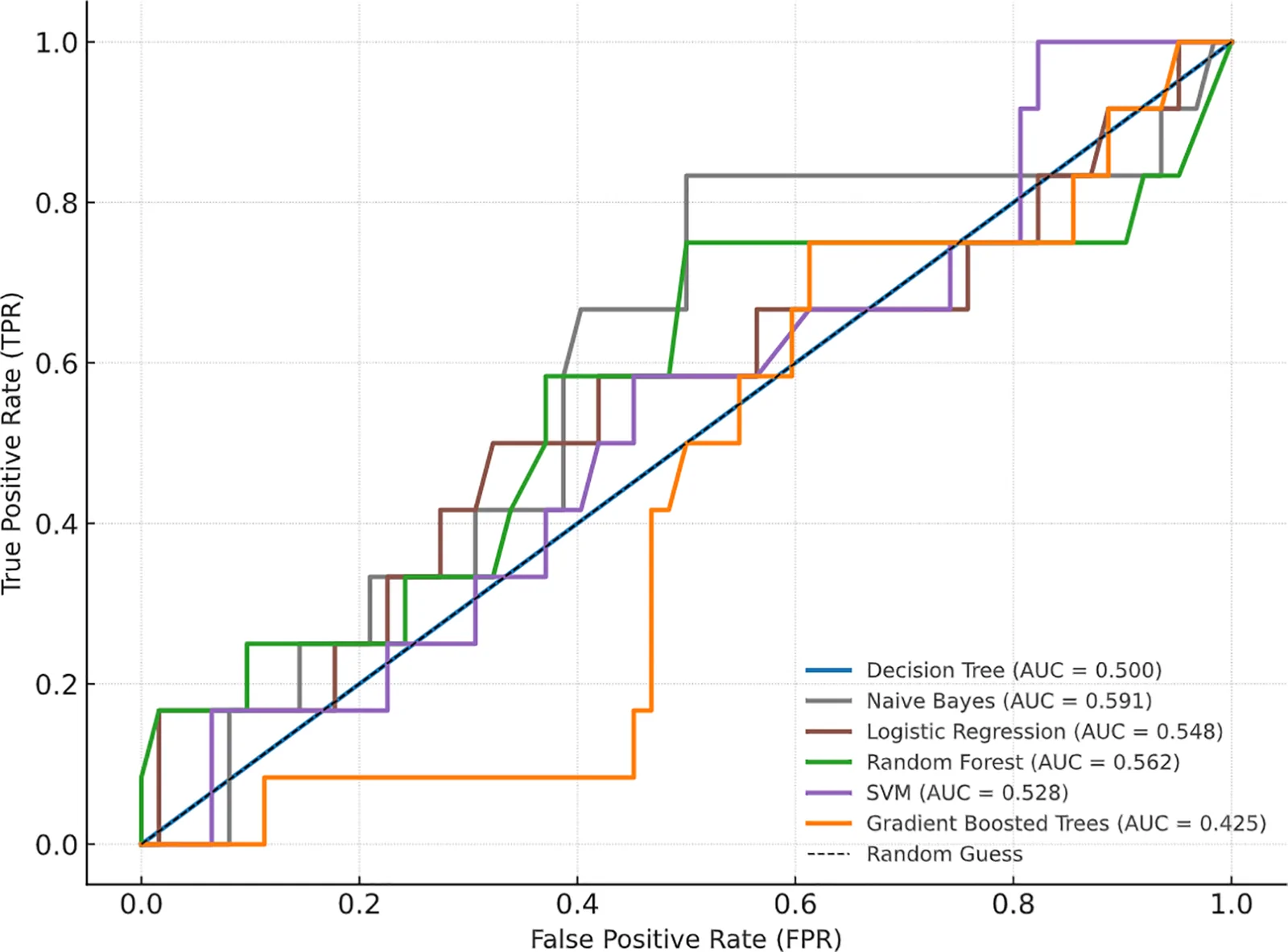
Receiver operating characteristic (ROC) curves of six classification models for job waiting time prediction.

The results show that all models exhibit relatively low discriminative ability, with AUC values ranging from 0.50 to 0.579. Support Vector Machine delivers the highest AUC value (0.579), followed by Random Forest (0.570), Gradient Boosted Trees (0.546), Logistic Regression (0.540), Naïve Bayes (0.507), and Decision Tree (0.500). The generally low AUC values indicate that the data used are not fully representative of the factors that influence the speed of obtaining employment. This phenomenon may be caused by two main factors: (1) most predictor variables are qualitative and relate to perceptions or subjective assessments, which makes them difficult to model statistically; and (2) the presence of class imbalance between the “Fast” and “Not Fast” categories, which leads to prediction bias in decision tree–based and boosting models.

Although the AUC values are relatively low, these results provide important insight that socio-psychological phenomena such as the speed of obtaining employment cannot be fully reduced to simple numerical patterns. This complexity suggests that other factors may play an important role that is not yet fully captured by the available data. Therefore, the ROC results serve as an empirical baseline for developing richer predictive models that integrate contextual and psychological variables in future research stages.

### 5.4 Relative model suitability under class imbalance

Based on the evaluation results, the Random Forest model shows relatively better metrics than the other five algorithms, with the highest Cohen’s Kappa (0.110), an AUC of 0.570, precision of 44.00%, and recall of 9.73%. However, these values remain weak in practical terms and only slightly above random guessing (AUC ≈ 0.57). Therefore, the model does not support predictive use at the individual level.

Algorithmically, the relative advantage of Random Forest lies in its ensemble learning (bagging) mechanism, which combines multiple decision trees to reduce variance and overfitting ( Breiman, 2001). This approach is suitable for the heterogeneous nature of tracer study data. Compared with Logistic Regression, which shows higher recall (31.86%) but low precision (17.39%), and Naïve Bayes or Decision Tree, which record zero recall, Random Forest demonstrates relatively better consistency, although its recall remains very low (9.73%).

The overall performance of the six models (AUC 0.500–0.579, Kappa ≤0.110) confirms the predictive limitations of the self-reported tracer study data used in this study, where perceptual variables provide limited information for distinguishing between the “Fast” and “Not Fast” classes. The models are not suitable as individual-level early warning systems, but they are useful as methodological evidence of the limits of employability predictive analytics and as a baseline for developing richer data sources, such as academic records, learning activities, and psychological factors.

Future research is recommended to apply class rebalancing techniques, such as SMOTE and cost-sensitive learning, and to include additional variables, as suggested by He and Garcia (2009). The combination of Random Forest for exploratory analysis and Logistic Regression for interpretative analysis remains relevant for aggregate diagnosis rather than deterministic prediction.

### 5.5 Logistic regression determinants of job waiting time

5.5.1. Major factors (Top 10 by AbsWeight).

Table 8 reports the top ten determinants of job waiting time derived from the logistic regression model, ranked by absolute weight (AbsWeight) and accompanied by the corresponding odds ratios (OR).

**Table 8.** Top ten determinants of job waiting time based on logistic regression.

No	Attributes	Weight	OR	AbsWeight
1	Internship = None	11.046	0.000	11.046
2	Demonstration = None	10.298	29.672	10.298
3	Course Quality = Poor	9.976	0.000	9.976
4	Research Project Participation = None	9.887	0.000	9.887
5	Field-Specific Expertise = Low	9.777	0.000	9.777
6	Use of Information Technology = Low	9.471	0.000	9.471
7	Teamwork = Moderate	1.459	0.232	1.459
8	Internship = Moderate	1.260	0.284	1.260
9	English Proficiency = Very High	1.146	3.146	1.146
10	Self-Development = Moderate	0.985	2.678	0.985

The logistic regression weight analysis identifies ten key factors that influence whether graduates obtain employment quickly or experience longer waiting time. The factor “Internship = None” shows the largest weight (Weight = 11.046; OR ≈ 0.000), indicating that the absence of internship experience significantly reduces graduates’ chances of securing a job quickly. Similar effects appear for “Course quality = Poor” (Weight = 9.976; OR ≈ 0.000) and “Research Project Participation = None” (Weight = 9.887; OR ≈ 0.000), which indicate that the quality of formal academic processes and research involvement are important factors in shortening job waiting time. In contrast, “Demonstration = None” has the largest positive weight (Weight = 10.298; OR ≈ 29.672), suggesting that students with limited involvement in non-academic activities tend to enter the labor market more quickly. In addition, “English Proficiency = Very High” (Weight = 1.146; OR ≈ 3.146) and “Self-Development = Moderate” (Weight = 0.985; OR ≈ 2.678) significantly increase the likelihood of faster employment. Other relevant factors include “Field-Specific Expertise = Low,” “Use of Information Technology = Low,” as well as “Teamwork = Moderate” and “Internship = Moderate,” which, although they have smaller weights, still contribute to graduates’ successful transition into the labor market.

Nevertheless, the very large Odds Ratio value (OR = 29.672) for the variable “Demonstration = None” needs to be interpreted with caution. Extreme OR values typically arise when a logistic regression model encounters a situation in which a variable almost perfectly separates outcome categories. This condition causes the logit coefficient to increase sharply, resulting in a disproportionate OR. In addition, such large values may also stem from estimation instability due to multicollinearity or the presence of outliers in the data. Therefore, this value is not interpreted as a literal effect size, but rather as an indication that the variable functions as a very strong separator within the data structure. Accordingly, interpretation in this study focuses more on the direction of relationships and statistical significance than on the absolute magnitude of the OR.

5.5.2. Moderate factors.

Table 9 summarizes the moderate determinants of job waiting time identified by the logistic regression model, including coefficient weights, odds ratios (OR), and absolute weights (AbsWeight).

**Table 9.** Moderate determinants of job waiting time identified by logistic regression.

No	Attributes	Weight	OR	AbsWeight
1	Ethics = High	0.582	1.789	0.582
2	Communication Skills = Moderate	0.478	1.612	0.478
3	Discussions = Moderate	0.426	1.531	0.426
4	Fieldwork = Moderate	0.590	1.804	0.590

In addition to dominant factors, the logistic regression analysis also identifies several variables with moderate effects on graduates’ job waiting time. These factors are characterized by absolute weight values ranging from 0.3 to 1.0, indicating meaningful contributions, although smaller than those of major factors such as internships or course quality.

For example, “Ethics = High” (Weight = 0.582; OR = 1.789) and “Communication Skills = Moderate” (Weight = 0.478; OR = 1.612) show that soft skills continue to play an important role in accelerating graduates’ transition into the labor market. Similarly, practical experiences such as “Discussions = Moderate” (Weight = 0.426; OR = 1.531) and “Fieldwork = Moderate” (Weight = 0.590; OR = 1.804) support job readiness, although they are not dominant factors. These findings confirm that shorter job waiting time is influenced not only by academic factors and internships but also by a balanced combination of practical experience and continuous soft skill development.

5.5.3. Lowest factors (AbsWeight = 0).

To distinguish predictors that provide no discriminative information, the results also report variable categories with null coefficients. Table 10 summarizes the lowest-impact factors (AbsWeight = 0), indicating categories that do not differentiate the Fast versus Not Fast outcomes in the fitted logistic regression model.

**Table 10.** Lowest-impact factors on job waiting time identified by logistic regression (absolute weight = 0).

No	Attributes	Weight	OR	AbsWeight
1	Communication Skills = High	0.000	1.000	0.000
2	Course Quality = Very High	0.000	1.000	0.000
3	Research Project Participation = High	0.000	1.000	0.000
4	Internship = Very High	0.000	1.000	0.000
5	Practical Training = High	0.000	1.000	0.000
6	Fieldwork = Very High	0.000	1.000	0.000
7	Discussions = Very High	0.000	1.000	0.000
8	Ethics = Very High	0.000	1.000	0.000
9	Demonstration = High	0.000	1.000	0.000
10	Field-Specific Expertise = High	0.000	1.000	0.000

The logistic regression analysis also identifies several variables with zero weights (Weight = 0; OR = 1), indicating that they do not have a significant effect on the speed at which graduates obtain employment. These variables include “Course Quality = Very High,” “Internship = Very High,” “Practical Training = High,” “Fieldwork = Very High,” “Discussions = Very High,” as well as personal attributes such as “Ethics = Very High,” “Communication Skills = High,” and “Field-Specific Expertise = High.” An odds ratio of 1 indicates that these extreme conditions do not differentiate graduates’ likelihood of belonging to the Fast or Not Fast category. This outcome may be caused by uneven data distribution, for example when the number of respondents in the “very high” category is relatively small, which prevents the model from detecting statistically significant differences.

Several variables show Odds Ratio values of 0 or OR = 1. This pattern indicates that, for certain variable categories, the model is unable to estimate coefficients in a stable manner. This condition typically occurs when a variable category appears only in one outcome class or when data variation is very limited, leaving the model with insufficient information to distinguish between graduates who obtain jobs quickly and those who do not. Numerically, this situation results in a weight of 0 or a constant OR value (0 or 1), indicating that the variable does not provide predictive information that can be reliably estimated from the available data.

5.5.4. Direction and magnitude of factor effects.

Table 11 summarizes the direction of relationships and the relative effect magnitude of the key logistic regression factors, grouping predictors with positive coefficients (accelerators of faster employment) and negative coefficients (barriers) alongside their corresponding odds ratios (OR).

**Table 11.** Direction and magnitude of factors affecting job waiting time.

Category	Attribute (Key examples)	Weight	OR	Interpretation
**Positive (Supports Faster Employment)**	Demonstration = None	10.298	29.672	No participation in demonstrations → dramatically increases the likelihood of faster employment
	English Proficiency = Very High	1.146	3.146	High English proficiency → increases the likelihood of faster employment by approximately three times
	Self-Development = Moderate	0.985	2.678	Moderate self-development → increases the likelihood of faster employment
	Fieldwork = Moderate	0.590	1.804	Moderate fieldwork experience → strengthens job readiness
	Ethics = High	0.582	1.789	Strong ethical standards → nearly doubles the likelihood of entering employment more quickly
**Negative (Hinders Faster Employment)**	Internship = None	−11.046	0.000	No internship experience → reduces the likelihood of faster employment to nearly zero
	Lectures = Poor	−9.976	0.000	Poor course quality → leads to longer job waiting time
	Research Projects Participation = None	−9.887	0.000	No research participation → decreases the likelihood of faster employment
	Field-Specific Expertise = Low	−9.777	0.000	Low competence → makes labor market absorption difficult
	Use of Information Technology = Low	−9.471	0.000	Weak IT skills → represent a significant barrier to employment

The logistic regression results also allow factors to be grouped based on the direction of their relationship with job waiting time. Factors with positive coefficients (Weight > 0) act as accelerators, while factors with negative coefficients (Weight < 0) function as barriers. Accelerating factors include “Demonstration = None at All” (Weight = 10.298; OR = 29.672), indicating that students who are not heavily involved in demonstration activities tend to obtain employment more quickly. Additional competencies such as “English Proficiency = Very High” (Weight = 1.146; OR = 3.146) and “Self-Development = Moderate” (Weight = 0.985; OR = 2.678) are also shown to shorten job waiting time. Similarly, “Fieldwork = Moderate” and “Ethics = High” contribute as supporting factors in accelerating graduates’ transition into the labor market.

In contrast, barriers to faster job attainment are indicated by large negative coefficients, such as “Internship = None at All” (Weight = −11.046; OR ≈ 0.000), “Course Quality = Poor” (Weight = −9.976; OR ≈ 0.000), and “Research Project Participation = None at All” (Weight = −9.887; OR ≈ 0.000). Low field-specific expertise and weak information technology proficiency also significantly reduce graduates’ chances of securing employment quickly. These findings show that shorter job waiting time is strongly influenced by a balance among academic factors, practical experience, and soft skills, while deficiencies in these fundamental aspects become the main barriers to graduate employability.

## 6. Discussion

### 6.1 Model performance evaluation

The findings indicate that the predictive performance of the classification models remains limited, suggesting that graduate job waiting time cannot be fully explained by the academic and competency-related variables captured in standard tracer study data. Rather than indicating methodological failure, this result highlights the multidimensional nature of graduate employability. Although tracer study variables reflect important aspects of learning experience, competencies, and soft skills, labor market transition is also shaped by broader psychological, social, and economic conditions that are not fully represented in institutional datasets. This interpretation is consistent with earlier studies showing that academic achievement and formal competencies explain only part of employment success, while adaptability, contextual readiness, and broader transition conditions also play important roles ( Schomburg, 2010).

At the same time, the comparative model results remain informative for institutional analysis. Logistic Regression shows relatively greater sensitivity to delayed-employment cases, whereas Random Forest provides the most balanced overall performance among the tested models. These findings suggest that tracer study data are more appropriate for aggregate-level diagnosis than for deterministic individual prediction. In practical terms, the models should not be interpreted as robust student-level early warning tools. Instead, their value lies in helping institutions identify broad patterns of graduate transition and in clarifying the limits of relying solely on standard tracer study indicators to explain employment outcomes. This interpretation is also consistent with the view that graduate job readiness depends on factors extending beyond university administrative variables, including social support, self-efficacy, and wider transition conditions ( Tomlinson, 2017).

From a higher education perspective, this result is important because it repositions tracer studies from a purely administrative reporting function toward a more analytical evaluative role. Even when predictive performance remains modest, tracer study data can still help universities examine whether learning experiences, competency development, and student support systems are sufficiently aligned with labor market transition outcomes. In this sense, the findings reinforce the importance of using tracer study evidence not merely to document outcomes, but to diagnose curriculum relevance and the effectiveness of educational provision ( Schomburg, 2010). When standard tracer indicators show limited predictive strength, the implication is not that tracer studies are unhelpful, but that they should be interpreted as one component of broader institutional evaluation.

This also has implications for higher education services. If employment transition is only partially captured by existing tracer-study indicators, universities need to strengthen the educational dimensions most likely to improve graduate readiness, particularly experiential learning opportunities and structured links between academic programs and the workplace. Earlier studies have shown that internships, industry-based projects, and service learning can strengthen graduates’ readiness by connecting academic knowledge with real-world demands ( Freudenberg et al., 2011). Accordingly, the principal value of the predictive analysis in this study lies not in identifying a highly accurate forecasting model, but in clarifying the analytical boundaries and practical usefulness of tracer-study-based evaluation for higher education improvement.

### 6.2 Factor analysis

The determinant analysis suggests that graduate job waiting time is shaped by the quality of educational experience, especially practical learning exposure, academic process quality, and competence formation. Graduates with no internship experience, weak engagement in academic processes, no research participation, limited field-specific expertise, and low information technology capability tend to face stronger barriers in entering employment. This pattern reinforces the view that higher education effectiveness cannot be reduced to degree completion alone; it also depends on whether institutions provide students with meaningful opportunities to build applied competence, work-related confidence, and familiarity with professional contexts. In this sense, internships, field-based learning, and research participation function not merely as supplementary activities, but as important bridges between academic learning and labor market expectations ( Yorke, 2006; Andrews & Higson, 2008; Kolb, 1984).

The findings also show that academic quality remains central to employability formation. Weak field-specific competence and low digital capability emerge as important barriers, suggesting that graduate transition into employment depends on both disciplinary mastery and readiness to function in technology-oriented work settings. This result supports the argument that higher education institutions need to strengthen not only curriculum content but also the applied relevance of teaching, learning design, and digital capability development. In labor markets characterized by technological change and increasing demands for adaptability, discipline-specific knowledge remains a core component of employability, while digital literacy operates as a baseline requirement rather than an optional advantage ( Yorke, 2006; van Laar et al., 2019).

Alongside academic and practical learning factors, the results underline the importance of transversal competencies. English proficiency, self-development, communication, ethics, discussions, and fieldwork all show positive associations with shorter job waiting time, although their strength varies. This indicates that graduate transition is supported not only by hard skills and practical exposure, but also by broader personal and interpersonal readiness. Such a pattern is consistent with employability scholarship that views job readiness as the product of interaction among academic competence, practical learning, soft skills, and continuing self-improvement ( Tomlinson, 2017; Clarke, 2018; Jackson, 2016). In this regard, self-development and English proficiency appear to function as differentiating assets that enhance graduates’ adaptability and competitiveness in increasingly complex labor markets, while communication and ethical orientation strengthen graduates’ capacity to function effectively in professional environments ( Candy, 2000; Fugate et al., 2004).

The findings also point to an important educational distinction between mere participation and meaningful learning intensity. Moderate teamwork and internship experience do not appear sufficient by themselves to produce a strong employability advantage, suggesting that practical learning must be designed with adequate quality, relevance, and integration into academic objectives. This interpretation is closely aligned with work-integrated learning research, which emphasizes that the value of workplace exposure depends not simply on duration, but on how well it is connected to structured learning, reflection, and disciplinary development ( Patrick et al., 2008; Jackson, 2015). In the same way, discussions and fieldwork appear to function as supportive experiences that strengthen graduate readiness when they are positioned as part of a broader competence-building process.

Some findings, however, should be interpreted cautiously. The association between low involvement in demonstration activities and faster employment may reflect category structure or estimation instability rather than a straightforward substantive effect. Likewise, variables with extreme coefficients or null effects indicate that not all categories are equally stable for literal interpretation. The finding that several “very high” categories do not differentiate employment outcomes may reflect uneven category distribution, limited variation, or the possibility that very strong performance among a small subgroup does not automatically translate into broad labor market advantage ( Haixiang et al., 2017). For this reason, the strongest contribution of the determinant analysis lies not in the exact magnitude of every coefficient, but in the broader pattern it reveals: graduate job waiting time is shaped by the interaction of practical learning, academic quality, competence formation, and supportive soft-skill development rather than by any single educational factor in isolation.

From an educational perspective, these findings imply that universities need to move toward a more integrated employability-oriented learning ecosystem. Practical experiences such as internships, field projects, and research engagement should be embedded more systematically into the curriculum; academic process quality should be maintained through stronger course delivery and competence-oriented teaching; and transversal capabilities such as communication, ethics, English proficiency, and self-development should be intentionally cultivated throughout the student journey rather than treated as peripheral outcomes. This also suggests that employability development should be inclusive and curriculum-wide rather than concentrated only among a small group of students with exceptionally high achievement. Studies on embedded employability similarly show that broad-based integration of career-related learning across the curriculum is more likely to strengthen graduate readiness at the population level than isolated interventions targeted only at selected high performers ( Crowne et al., 2020; Small et al., 2018; Siivonen et al., n.d.). In this sense, higher education services need to be understood as a connected ecosystem in which curriculum, practical learning, digital capability, and student development initiatives jointly shape graduates’ readiness for labor market transition. Overall, the findings suggest that graduate job waiting time is best understood as the outcome of an integrated educational process in which practical learning, academic quality, and transversal capability development jointly shape labor market transition.

### 6.3 Implications for higher education services

The findings of this study have important implications for higher education services and curriculum development. First, practical learning should be positioned as a core component of graduate preparation rather than as a supplementary requirement. Internships, research participation, field-based learning, and other applied academic experiences appear to function as important bridges between university learning and labor market expectations. This implies that higher education institutions need not only to provide access to such experiences, but also to ensure their relevance, quality, supervision, and alignment with academic objectives. In this regard, work-integrated and high-impact learning experiences can be understood as structured educational mechanisms that strengthen graduate readiness for employment transition ( Jackson, 2015).

Second, the results reinforce the importance of maintaining academic process quality as a central foundation of employability development. Weak course quality, limited field-specific expertise, and low information technology capability appear to reduce graduates’ readiness for labor market transition. This suggests that curriculum improvement should not be limited to updating course content, but must also address teaching quality, applied learning design, and competence-oriented delivery. In labor markets characterized by technological change and increasing demands for adaptability, graduate employability is shaped by the interaction of human capital, individual attributes, and contextual opportunity structures, while digital capability increasingly operates as a baseline condition for effective participation in knowledge-intensive work ( Clarke, 2018; van Laar et al., 2019).

Third, the findings highlight the importance of transversal and developmental competencies in supporting graduate transition. Communication, ethics, English proficiency, and self-development should not be treated as peripheral attributes, but as part of the broader employability ecosystem that universities intentionally cultivate. These competencies need to be embedded across the curriculum, co-curricular activities, and student development services so that employability is developed progressively throughout the student journey rather than addressed only at the point of graduation. This interpretation is consistent with scholarship that conceptualizes employability as a broader configuration of graduate capital rather than a narrow set of technical skills, and with the argument that lifelong learning orientation remains a core mandate of higher education ( Tomlinson, 2017; Candy, 2000; Clarke, 2018).

Finally, the findings suggest that tracer study systems should be used more strategically as instruments of educational improvement. Their value lies not merely in reporting graduate destinations, but in helping institutions identify structural strengths and weaknesses in curriculum relevance, practical learning provision, and student readiness. Used in this way, tracer studies can support more evidence-based higher education governance and more responsive service design, especially when interpreted as tools for institutional diagnosis rather than as precise systems for individual-level prediction. This is consistent with tracer study guidance that positions graduate surveys as tools for improving education and for examining the relevance of education and training to early career transition.

### 6.4 Contribution, limitations, and future directions

This study makes both empirical and practical contributions to the higher education literature. Empirically, it shows that tracer study data can provide meaningful institutional insight into graduate job waiting time, even when their predictive value remains limited for individual-level classification. More specifically, the study contributes by linking comparative model evaluation with the identification of education-related dimensions associated with faster and slower transition into employment. In doing so, it extends the use of tracer study data beyond descriptive reporting and demonstrates their relevance for institutional diagnosis, especially in relation to curriculum relevance, practical learning exposure, competence formation, and graduate readiness.

The study also makes a practical contribution by clarifying which aspects of higher education services appear most closely connected to employment transition. The findings suggest that practical learning, academic process quality, field-specific competence, digital capability, and transversal competencies should be understood as interconnected dimensions of employability support. This provides a more substantive basis for curriculum refinement and for the strengthening of employability-oriented student development strategies in higher education.

At the same time, several limitations should be acknowledged. The dataset is derived from a single faculty, which limits the broader generalizability of the findings across institutions, disciplines, and labor market contexts. In addition, most variables are based on self-reported tracer study responses, which may not fully capture the complexity of graduate capability and employment transition. Class imbalance also constrains model discrimination, and some category estimates appear sparse or unstable, meaning that not all coefficients should be interpreted literally.

These limitations point to several directions for future research. Subsequent studies should expand institutional coverage and compare results across faculties, universities, and fields of study. Future work would also benefit from integrating richer non-academic variables, including psychological, social, and contextual dimensions that are often absent from standard tracer datasets. In addition, stronger linkage between tracer data and curriculum design, work-integrated learning experiences, academic records, and digitally mediated skill development would allow graduate transition to be examined more comprehensively while preserving the practical value of tracer-study-based evaluation.

## 7. Conclusion

This study shows that tracer study data can provide meaningful institutional insight into graduate job waiting time, although their predictive value remains limited for individual-level classification. The comparison of six classification models indicates that high accuracy alone can be misleading under class imbalance, as several models performed poorly in identifying graduates with delayed employment. Among the evaluated models, Logistic Regression was relatively more useful for detecting the minority class and for interpreting factor relationships, whereas Random Forest provided the most balanced overall performance. However, the overall results indicate that none of the models demonstrated sufficiently strong discriminative power to support precise individual-level prediction.

More importantly, the study identifies several education-related factors associated with employment transition. Delayed employment is more strongly associated with the absence of internship experience, weak academic process quality, lack of research participation, limited field-specific expertise, and low information technology capability. In contrast, faster transition into employment is associated with stronger English proficiency, self-development, communication, ethics, discussions, and fieldwork. These findings suggest that graduate job waiting time is shaped not by academic variables alone, but by the interaction of practical learning, competence formation, academic quality, and transversal capability development.

From a higher education perspective, the results imply that tracer studies are most valuable when used as tools for institutional diagnosis rather than as deterministic prediction systems. Their main contribution lies in helping universities identify structural strengths and weaknesses in graduate preparation, particularly in relation to curriculum relevance, practical learning opportunities, digital capability, and employability-oriented student development. Accordingly, higher education institutions need to strengthen structured work-integrated learning, maintain the quality of academic processes, and embed transversal competencies more systematically across the student journey.

This study has several limitations, including class imbalance, reliance on self-reported variables, and the focus on a single faculty, which limits broader generalizability. Future research should therefore expand institutional coverage, incorporate richer non-academic variables, and integrate additional data sources capable of capturing psychological, social, and labor market dimensions more fully. Overall, the study concludes that the principal value of tracer study data lies in supporting evidence-based higher education improvement and graduate employability support rather than in serving as stand-alone predictive instruments.

## Compliance with ethical standards

Ethical Approval: This study received ethical approval from the Faculty of Administrative Sciences, Universitas Brawijaya (Approval No. 00547/UN10.F0301/B/PP/2026, Date: 12 Januari 2026). All data were processed in anonymized form. Informed consent was obtained from participants at the time of the tracer study, and the research was conducted in accordance with applicable institutional guidelines.

Informed consent: Informed consent was obtained in written electronic form at the beginning of the online tracer study survey. Participants indicated consent by selecting an ‘I agree’ option before proceeding. No identifying information was collected for research purposes, and data were analyzed in anonymized form.

## Data Availability

The underlying data for this study are available from Figshare: Tracer Study Data for Graduate Job Waiting Time Analysis (2021–2023). https://doi.org/10.6084/m9.figshare.32786688 ( Hermawan et al., 2026). The dataset contains anonymized tracer study records used to examine graduate job waiting time and to support evidence-based improvement of higher education services. The data are available under the Creative Commons Attribution 4.0 International License (CC BY 4.0).
