# Supplementary material for: Data-Driven Policy: An Analysis of Graduate Job Waiting Time and Its Implications for Higher Education Services
Source: F1000Res. 2026 Sep 8;15:1135. Originally published 2026 Jul 11. [Version 2] doi: 10.12688/f1000research.185630.2 (PMC13648953; doi:10.12688/f1000research.185630.2)
Supplement: Peer review history

# Reviewer report: Reviewer response for version 2

**DOI:** 10.5256/f1000research.209675.r519190

**Published:** 2026-09-11

**Authors:** Heap Yih (John) Chong

**Affiliations:** 1 Nanjing Audit University, Nanjing, China

I have no further comments.

Is the work clearly and accurately presented and does it cite the current literature?

Partly

If applicable, is the statistical analysis and its interpretation appropriate?

Yes

Are all the source data underlying the results available to ensure full reproducibility?

Yes

Is the study design appropriate and is the work technically sound?

Yes

Are the conclusions drawn adequately supported by the results?

Yes

Are sufficient details of methods and analysis provided to allow replication by others?

Partly

Reviewer Expertise:

Social science research

I confirm that I have read this submission and believe that I have an appropriate level of expertise to confirm that it is of an acceptable scientific standard.

# Reviewer report: Reviewer response for version 2

**DOI:** 10.5256/f1000research.209675.r519188

**Published:** 2026-09-10

**Authors:** Eka Desy Purnama

**Affiliations:** 1 Krida Wacana Christian University, Jakarta, Jakarta, Indonesia

I have reviewed the revised version of this manuscript and appreciate the authors’ efforts in addressing the concerns raised in the previous review. The revisions have improved the methodological clarity, interpretation of the classification results, discussion of class imbalance and model performance, as well as the presentation of the study’s limitations and implications for higher education services. The manuscript now provides a clearer distinction between the use of tracer study data for institutional evaluation and its limitations for individual-level prediction. Overall, I consider that the authors have satisfactorily addressed my previous comments and that the manuscript is now sufficiently clear, coherent, and methodologically sound. Therefore, I recommend Approved for this revised version.

Is the work clearly and accurately presented and does it cite the current literature?

Yes

If applicable, is the statistical analysis and its interpretation appropriate?

Yes

Are all the source data underlying the results available to ensure full reproducibility?

Partly

Is the study design appropriate and is the work technically sound?

Yes

Are the conclusions drawn adequately supported by the results?

Yes

Are sufficient details of methods and analysis provided to allow replication by others?

Partly

Reviewer Expertise:

Business, Economic Management, and Financial Management

I confirm that I have read this submission and believe that I have an appropriate level of expertise to confirm that it is of an acceptable scientific standard.

# Reviewer report: Reviewer response for version 1

**DOI:** 10.5256/f1000research.204890.r508425

**Published:** 2026-08-31

**Authors:** Heap Yih (John) Chong

**Affiliations:** 1 Nanjing Audit University, Nanjing, China

This study analyzes tracer study data from 1,280 graduates using six classification models to examine job waiting time, finding limited predictive power for individual-level forecasting. Below are the comments:

 - The Abstract should be rewritten into a one long paragraph if the format allows.

 - The study is framed as quantitative but includes a qualitative phase (Table 5) with no methodology, analysis, or findings—this section should either be removed or fully integrated.

 - All models show poor discriminative power (AUC ≤ 0.579, Kappa ≤ 0.110), confirming they are unsuitable for individual-level prediction despite being useful for institutional diagnosis.

 - The "Fast" employment category is defined inconsistently as both "≤ 6 months" and "< 6 months" across sections—this needs standardization.

 -"Significant" is used without p-values or confidence intervals; extreme OR values (e.g., 29.672) likely reflect estimation instability and should be interpreted with more caution.

 - Data from a single faculty restricts broader applicability; recommendations should be framed as institutional considerations rather than broad policy conclusions.

Is the work clearly and accurately presented and does it cite the current literature?

Partly

If applicable, is the statistical analysis and its interpretation appropriate?

Yes

Are all the source data underlying the results available to ensure full reproducibility?

Yes

Is the study design appropriate and is the work technically sound?

Yes

Are the conclusions drawn adequately supported by the results?

Yes

Are sufficient details of methods and analysis provided to allow replication by others?

Partly

Reviewer Expertise:

Social science research

I confirm that I have read this submission and believe that I have an appropriate level of expertise to confirm that it is of an acceptable scientific standard, however I have significant reservations, as outlined above.

# Reviewer report: Reviewer response for version 1

**DOI:** 10.5256/f1000research.204890.r508419

**Published:** 2026-08-11

**Authors:** Eka Desy Purnama

**Affiliations:** 1 Krida Wacana Christian University, Jakarta, Jakarta, Indonesia

The manuscript addresses a relevant issue and offers useful implications for graduate employability and higher education services. However, several points should be clarified before the article can be fully approved.

 The principal concern relates to the research design. The manuscript consistently presents the study as a quantitative analysis, yet Section 4.5 introduces a qualitative phase and provides a table of qualitative informants. No information is given regarding interview procedures, data analysis, or qualitative findings. This is more than a minor editorial inconsistency, as it leaves the reader uncertain about whether the study is quantitative or mixed methods. The authors should either remove the qualitative component entirely or explain and report it properly throughout the manuscript.

 A few further points may also be considered. The objectives section would benefit from presenting the research questions individually rather than embedding them in one extended paragraph. This would make the relationship between the research questions, results, and discussion easier to follow.

 The explanation for the selection of the classification algorithms could also be expanded. While the manuscript discusses Naïve Bayes, Decision Tree, and Logistic Regression, the reasons for including Random Forest, Support Vector Machine, and Gradient Boosted Trees are not equally clear.

 Finally, the implications should remain closely aligned with the scope of the evidence. Since the dataset is limited to one faculty and the models have relatively weak discriminative performance, the recommendations are more appropriately framed as institutional considerations than as broadly applicable policy conclusions.

 The manuscript has value, but the inconsistency surrounding the study design must be resolved to ensure methodological transparency. On this basis, the manuscript is recommended as Approved with Reservations.

Is the work clearly and accurately presented and does it cite the current literature?

Yes

If applicable, is the statistical analysis and its interpretation appropriate?

Yes

Are all the source data underlying the results available to ensure full reproducibility?

Partly

Is the study design appropriate and is the work technically sound?

Yes

Are the conclusions drawn adequately supported by the results?

Yes

Are sufficient details of methods and analysis provided to allow replication by others?

Partly

Reviewer Expertise:

Business, Economic Management, and Financial Management

I confirm that I have read this submission and believe that I have an appropriate level of expertise to confirm that it is of an acceptable scientific standard, however I have significant reservations, as outlined above.

# Response

**Published:** 2026-08-31

**Authors:** Hermawan Hermawan

**Affiliations:** Universitas Brawijaya, Malang, East Java, Indonesia

We sincerely thank the reviewer for the careful evaluation of our manuscript and for the constructive suggestions. We have revised the manuscript accordingly to improve methodological clarity and consistency.

 Comment 1 - Research design

 Section 4.5 has been revised to clarify that the study is entirely quantitative. References to a qualitative phase and qualitative informants have been removed.

 Comment 2 - Research questions

 The research questions are now presented separately as RQ1–RQ4 to improve clarity and alignment with the results and discussion.

 Comment 3 - Selection of classification algorithms

 Section 4.3 has been expanded to provide clearer justification for the inclusion of Random Forest, Support Vector Machine, and Gradient Boosted Trees.

 Comment 4 - Implications

 The implications have been revised to emphasize context-specific institutional considerations, with clearer acknowledgment of the single-faculty scope and the limited discriminative performance of the models.

 We appreciate the reviewer’s valuable feedback, which has helped strengthen the clarity and methodological transparency of the manuscript.

# Reviewer report: Reviewer response for version 1

**DOI:** 10.5256/f1000research.204890.r508417

**Published:** 2026-08-10

**Authors:** Muhammad Khairul Anwar

**Affiliations:** 1 State University of Surabaya, Surabaya, Indonesia

The manuscript addresses a relevant issue and is generally clear, well structured, and appropriately cautious in interpreting the limited predictive performance of tracer study data. The following minor revisions are suggested:

 1.    The definition of the “Fast” employment category should be standardized. Some sections use a waiting time of six months or less, while Table 2 appears to use less than six months. The same threshold should be applied throughout the manuscript.

 2.    A few methodological details would improve clarity and reproducibility. The authors should report the number of cases in each employment category, the number of folds used in cross-validation, and how missing values and categorical variables were handled.

 3.    The interpretation of the logistic regression results should be slightly more cautious. The term “significant” should only be used when supported by p-values or confidence intervals. Otherwise, wording such as “associated with” would be more appropriate.

 4.    A final language and consistency check is recommended. Some points regarding internships, academic quality, and digital skills are repeated across the Discussion and Conclusion.

 These comments mainly concern clarity and reporting consistency and do not affect the main findings or contribution of the study. The manuscript can therefore be recommended as Approved.

Is the work clearly and accurately presented and does it cite the current literature?

Yes

If applicable, is the statistical analysis and its interpretation appropriate?

Yes

Are all the source data underlying the results available to ensure full reproducibility?

Yes

Is the study design appropriate and is the work technically sound?

Yes

Are the conclusions drawn adequately supported by the results?

Yes

Are sufficient details of methods and analysis provided to allow replication by others?

Partly

Reviewer Expertise:

Public Administration, Policy, and Governance

I confirm that I have read this submission and believe that I have an appropriate level of expertise to confirm that it is of an acceptable scientific standard.

# Response

**Published:** 2026-08-31

**Authors:** Hermawan Hermawan

**Affiliations:** Universitas Brawijaya, Malang, East Java, Indonesia

We sincerely thank the reviewer for the positive assessment of our manuscript and for the constructive suggestions provided. We have carefully addressed all comments to improve the clarity, methodological reporting, consistency, and interpretation of the manuscript. The employment category definition has been standardized, relevant methodological details have been clarified, the interpretation of the logistic regression results has been revised using more cautious wording, and the manuscript has undergone a final language and consistency check to reduce repetition. We appreciate the reviewer’s valuable feedback and recommendation for approval.
